# Fungivorous mites enhance the survivorship and development of stingless bees even when exposed to pesticides

**DOI:** 10.1038/s41598-022-25482-x

**Published:** 2022-12-05

**Authors:** Annelise S. Rosa-Fontana, Adna Suelen Dorigo, José Bruno Malaquias, Jéssica K. S. Pachú, Roberta C. F. Nocelli, Simone Tosi, Osmar Malaspina

**Affiliations:** 1 State University of Sao Paulo Júlio de Mesquita Filho, Rio Claro, SP Brazil; 2 Escola Superior de Agricultura “Luiz de Queiroz”, University of Sao Paulo, Piracicaba, SP Brazil; 3 Centre of Agrarian Science, Federal University of Sao Carlos, Araras, SP Brazil; 4 Department of Agricultural, Forest, and Food Sciences, University of Torino, Grugliasco, Italy

**Keywords:** Animal behaviour, Entomology, Ecology, Biodiversity, Conservation biology

## Abstract

Stingless bees are the largest group of eusocial bees in the world. They play an essential role as crop pollinators and have been considered for inclusion in pesticide risk assessments (RAs). Beyond the mutualism involving stingless bee larvae and fungi, the fungivorous mite *Proctotydaeus (Neotydeolus) alvearii* proved to be interesting for studies of associations with stingless bees. Their presence is related to colony strength and health, showing a permanent-host-association level. Here, we tested whether the coexistence with *P.* (*N.*) *alvearii* affects stingless bee larvae survivorship and development, including when fed pesticide-dosed food. We chose dimethoate, the reference standard for toxicity tests, and thiamethoxam, widely used in neotropical crops and listed to be reassessed in RAs. Bees associated with the mites showed higher larval survivorship rates, even in the dosed ones, and revealed changes in the developmental time and body size. Our study represents the first approach to stingless bee responses to the coexistence of fungivorous mites inside brood cells, leading us to believe that these mites play a beneficial role in stingless bees, including when they are exposed to pesticides.

## Introduction

In recent decades, multiple interacting stressors, such as climate change, land use intensification, invasive species, genetically modified crops, parasites, habitat losses, lack of flowers and pesticide use, have been linked to pollinator losses worldwide^1–5^. This concern encompasses bee populations in general; thus, it is prudent to find alternatives to keep the bees strong and healthy. Stingless bees (Apidae: Meliponini), which are of neotropical distribution, are the major group of eusocial bees in the world. They live in perennial colonies that typically contain dozens to thousands of individuals^6^. Interactions involving stingless bees and microorganisms are essential: bacteria, fungi and yeasts play fundamental roles related to nutrition and protection against harmful microorganisms^7–14^. The ingestion requirements of the stingless bee *Scaptotrigona* sp. larvae for the fungus *Zygosaccharomyces* sp. have been reported; however, the microbiota in brood combs is not limited to this required fungus^11,12,15^.

In addition to these mutualistic associations, mites appear to be interesting organisms to be included in studies on this subject. Flechtmann and Camargo^16^ described the presence of the fungivorous mite *Neotydeolus therapeutikos* (Prostigmata: Tydeidae) in brood cells of the stingless bee *Scaptotrigona postica*, reporting significantly lower larval mortality rates when the mites were present. Currently classified as a subgenus, *Neotydeolus* (the only subgenus of *Proctotydaeus* containing bee-associated mites) is found in the neotropical region, where it has been reported on stingless bees and the genera *Melipona*, *Partamona*, and *Scaptotrigona*. The host association level is permanent, as these mites cannot live without bees or wasps^16^. The mites are not always in the nests, but when they are present, the colonies are strong and healthy.

We investigated whether the presence of *Proctotydaeus* (*Neotydeolus*) *alverii fungivorous* mite species living inside brood cells interferes with the survivorship and developmental biological parameters of *S. postica* larvae in vitro reared, exposed or not exposed to pesticides. We hypothesized a beneficial role of the mites on stingless bee survivorship. We further investigated whether fungivorous mites can protect bees from pesticide stress. Our results provide information for the development of further investigations on these organisms as alternatives to improve bee health and to protect bees against pesticides. We selected for this investigation the stingless bee *S. postica*, which has been studied for inclusion as a model organism in risk assessments (RAs) in the neotropics^17–19^. We used thiamethoxam, a neonicotinoid that is commonly used in neotropical crops, as an active ingredient, and dimethoate, the reference standard for toxicity tests.

We showed that bees associated with the mites presented higher larval survivorship rates, even in the dosed bees, and revealed changes in the developmental time and body size. Our study represents the first demonstration of stingless bee interactions with fungivorous mites. Our findings show that mites play a beneficial role in stingless bee health and tolerance to pesticides.

## Results

### Identification and behavioural observations of the mites

**Figure 1:**
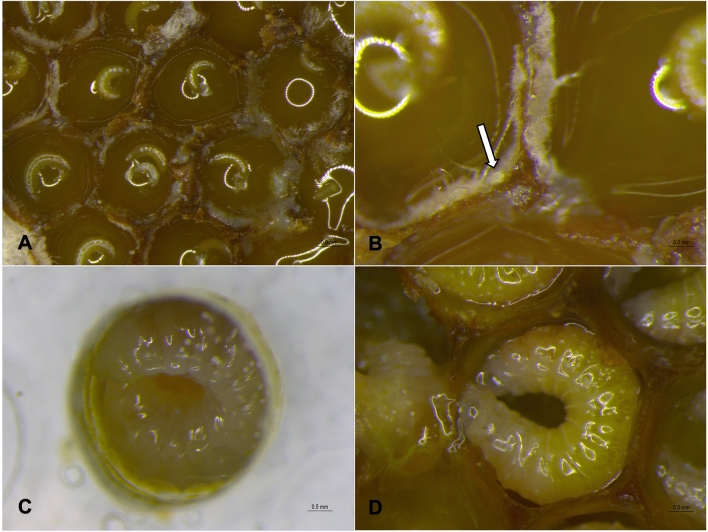
*Scaptotrigona postica* and its beneficial mites. (**a**) Brood cells in the brood combs of *S. postica*, (**b**) Newly-uncapped brood cell: *P.* (*N*.) *alvearii* (indicated by the arrow) feeding on the fungi inside *S. postica* brood cells, (**c**) *S. postica* larvae completely fed in the in vitro rearing plates, with mites no longer seen, and (**d**) *S. postica* larvae completely fed in the natural system, with mites no longer seen.

The mite species was identified as *Proctotydaeus* (*Neotydeolus*) *alvearii* Rosa, André and Flechtmann^20^ (Prostigmata: Tydeidae). Several aspects of the behaviour of *P*. (*N.*) *alvearii* were observed in newly uncapped brood combs: walking in the cells; approaching and touching the bee eggs; and feeding on fungi or on larval food before and during larval feeding (Fig. 1a–b and Supplementary Video 1). Their behaviour of the mites is consistent with a cleaning task that is performed inside brood cells. The mites are seen walking on developing larvae and on larval food, seemingly feeding on the fungus.

### General survivorship of stingless bees

**Figure 2:**
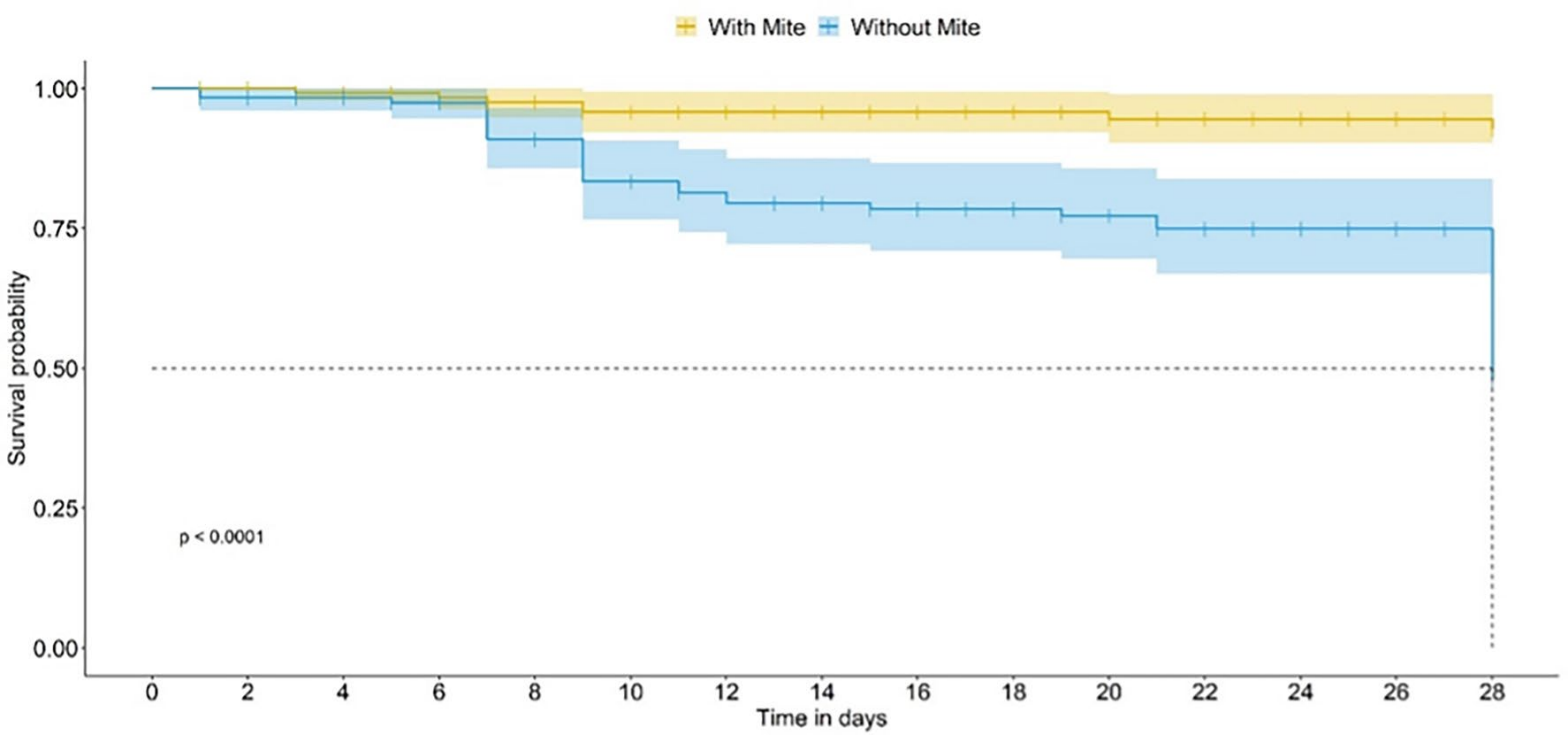
*Scaptotrigona postica* survivorship in the presence (with mites) and in the absence (without mites) in the control bioassay. The dashed line indicates the estimated median life span for bees in the presence and absence of the mite.

**Figure 3:**
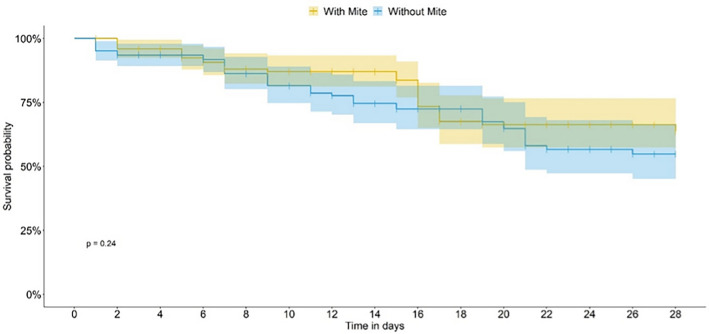
*Scaptotrigona postica* survivorship in the presence (with mites) and in the absence (without mites) in the thiamethoxam bioassay (TMX).

**Figure 4:**
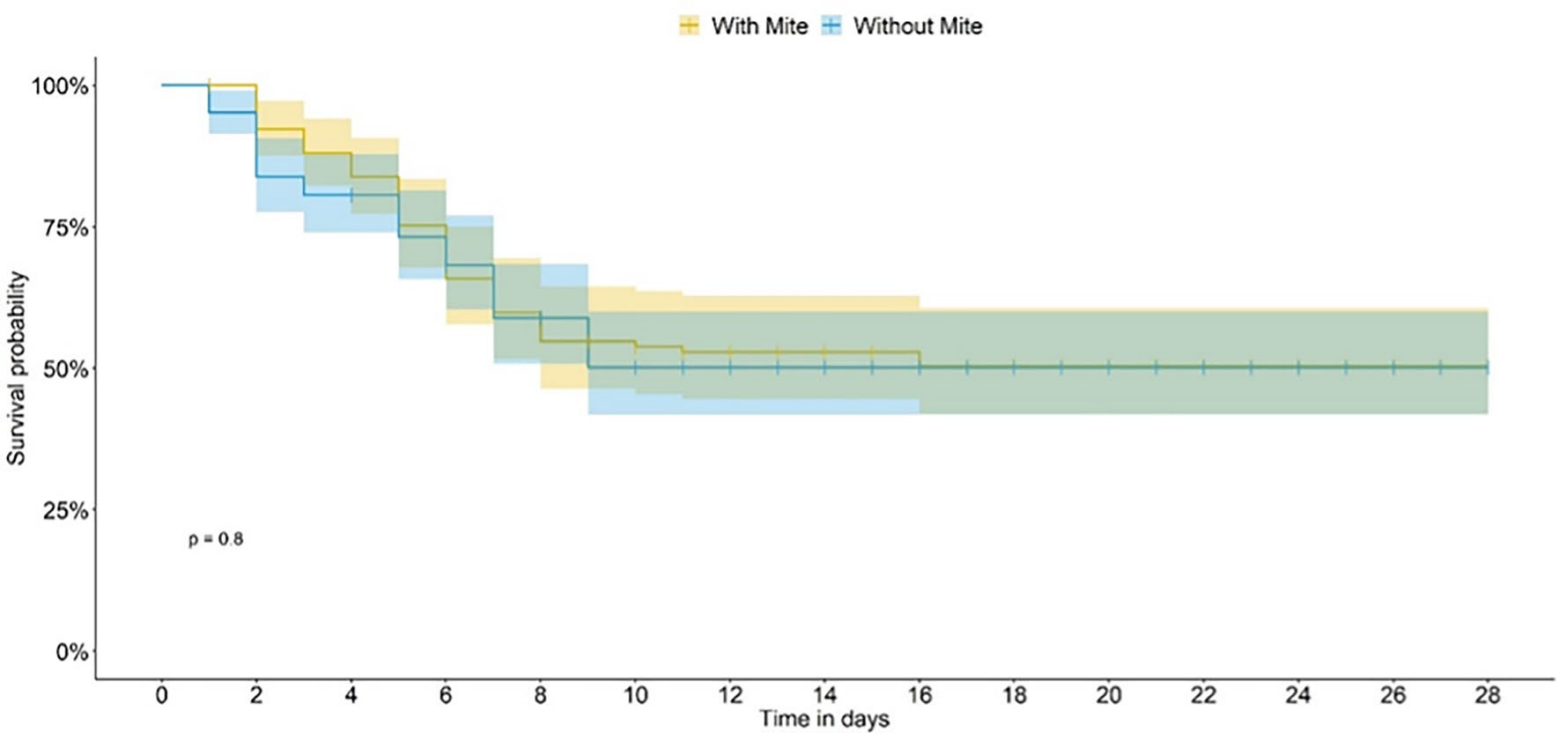
*Scaptotrigona postica* survivorship in the presence (with mites) and in the absence (without mites) in the dimethoate bioassay (DIM).

On food not treated with pesticide (control—CONT bioassay), a significant difference between bee survival curves in the presence or absence of the mite was observed, as of the seventh day of the introduction of the mite in the experimental units (Generalized linear model (GLM); *P* < 0.0001; Fig. 2), in both cases for a median bee lifetime of 28 days. When the bees were fed thiamethoxam (TMX-dosed), the presence of mites did not interfere with the survivorship curves (Figs. 3 and 4). When the bees were fed on dimethoate (DIM-dosed), survivorship was greatly reduced, with no survivorship when mites were absent; the time-response of the bees did not allow us to estimate the median lifetime in both TMX and DIM.

### Survivorship in each developmental phase

The larvae were favoured by the presence of mites in all bioassays: whereas the survivorship rates in CONT with mites was 91%, without them the survivorship rate was 39% (GLM; *F* = 32.06, *P* = 0.004; Table 1). The same parameter for TMX was 69% with mites, against 35% without them (GLM; *F* = 6.9273, *P* = 0.05; Table 1). In the DIM treatment, there was no larval survivorship when mites were absent; however, in their presence, survivorship was almost 8% (Table 1).

**Table 1:** Summary of *Scaptotrigona postica* survivorship in each development phase.

	LARVA			PUPA			POSTEMBRYONAL IMMATURE		
Bioassay	CONT	TMX	DIM	CONT	TMX	DIM	CONT	TMX	DIM
Mite	91.49 ± 04.61a (N = 59)	69.56 ± 04.79a (N = 59)	07.76 ± 02.76 (N = 58)	96.25 ± 01.90a (N = 54)	59.76 ± 09.41a (N = 41)	50.00 ± 35.31 (N = 3)	87.98 ± 04.74a (N = 59)	42.45 ± 09.00a (N = 59)	02.50 ± 02.50 (N = 58)
No mite	39.25 ± 05.74b (N = 58)	35.96 ± 11.33b (N = 59)	NA (N = 60)	63.70 ± 15.19b (N = 23)	84.84 ± 15.15a (N = 21)	NA (N = 0)	24.07 ± 05.84b (N = 58)	27.19 ± 03.62a (N = 59)	NA (N = 60)
*df; F*; > *P*	df = 1; F = 32.06; *P* = 0.004	df = 1; F = 6.9273; *P* = 0.050	–	df = 1; F = 6.77; *P* = 0.05	df = 1; F = 1.50; *P* = 0.2878	–	df = 1; *F* = 45.27; *P* = 0.0025	df = 1;	–
								*F* = 2.5943; *P* = 0.1825	

Survivorship (%) (Mean ± SE) of bees in each development phase (larva, pupa and postembryonal immature) in the presence (Mite) and in the absence (No mite) of mites on diets treated with thiamethoxam, dimethoate and untreated (respectively TMX, DIM and CONT bioassays). Means were contrasted by the F test from a generalized linear model of the quasibinomial type (α = 005). *N* Initial number of individuals, *NA* No applicable.

For pupae, in the CONT bioassay, survivorship with mites was significantly higher (96%) than survivorship with no mites (63%) (GLM; *F* = 6.77, *P* = 0.05; Table 1). Mites did not significantly affect the pupae survivorship rates in the TMX bioassay (Table 1). As also found for larvae, in the DIM bioassay, pupae survived only when mites were present (50%) (Table 1).

The survivor rates of postembrionary immatures in the CONT bioassay with mites were higher (87%) than the survivorship rates without mites (24%) (GLM; *F* = 45.27, *P* = 0.0025; Table 1). The mean survivorship of the bees in the TMX bioassay ranged between 27 and 42%, with no significant difference between treatments (Table 1). In the DIM bioassay, some surviving bees were found (2%) (Table 1).

### Developmental time of each stage

For larvae, a significant difference between treatments was observed in the CONT bioassay, with the time being longer in the absence of mites (GLM; *F* = 87.59, *P* = 0.0007; Table 2). Mites did not significantly affect the larval survivorship rates in the TMX bioassay (Table 2). In the DIM bioassay, the median duration of the surviving larvae (when mites were present) was 17 days (Table 2).

**Table 2:** Summary of *Scaptotrigona postica* development time in each stage. Duration (%) (Weighted Mean ± SE) of each development phase (larva, pupa and newly emerged) in the presence (Mite) and in the absence (No mite) of mites and exposed to thiamethoxam (TMX) and dimethoate (DIM) and the control group (CONT).

Bioassay	LARVA			PUPA			POSTEMBRYONAL IMMATURE		
	CONT	TMX	DIM	CONT	TMX	DIM	CONT	TMX	DIM
Mite	17.49 ± 0.20b (N = 54)	17.61 ± 0.21a (N = 44)	17.50 ± 0.50 (N = 3)	11.50 ± 0.20a (N = 54)	11.38 ± 0.21a (N = 41)	12.00 ± 0.00 (N = 3)	29,00 ± 0.00 b (N = 52)	29,00 ± 0.00b (N = 25)	19,50 ± NA (N = 1)
No mite	19.60 ± 0.09a (N = 27)	17.98 ± 0.50a (N = 31)	NA (N = 0)	09.67 ± 0.14b (N = 23)	11.55 ± 0.44a (N = 21)	NA (N = 0)	29,28 ± 0,06 a (N = 14)	29,53 ± 0,07 a (N = 16)	NA (N = 0)
*F*; > *P*	df = 1; *F* = 87.59; *P* = 0.0007	df = 1; *F* = 0.44; *P* = 0.5396		df = 1; *F* = 53.47; *P* = 0.0018	df = 1; *F* = 0.10; *P* = 0.7570		df = 1; *F* = 40.96; *P* = 0.0001	df = 1; *F* = 56.82; *P* = 0.0016	

Averages followed by the same letters do not differ by t test (LSD—significant minimum difference) with Bonferroni protection (α = 0,05). *N* Number of individuals analysed, *NA* No applicable.

For pupae, a significant difference between treatments was observed in the CONT bioassay, but time was longer in the presence of mites (GLM; *F* = 53.47, *P* = 0.0018; Table 2). Mites did not significantly affect the pupae survivorship rates in the TMX bioassay (Table 2). In the DIM bioassay, the median duration of the surviving pupae (when mites were present) was 12 days (Table 2).

In pooling durations of larvae and pupae for each bee (postembryonal immatures), significant differences were observed for the CONT (GLM; *F* = 40.96, *P* = 0.0001; Table 2) and the TMX (GLM; *F* = 56.82, *P* = 0.0016; Table 2) bioassays, with the time being longer in the absence of mites. Comparisons were not made in the DIM bioassay, but the duration of the surviving bees (when mites were present) was 19 days (Table 2).

### Morphometry

In the CONT bioassay, bees had a significantly wider head (HW) (GLM; *F* = 132.24, *P* = 0.0001; Table 3) and shorter intertegular distance (ID) (GLM; *F* = 7.19, *P* = 0.0147; Table 3) when mites were present. In the TMX bioassay, bees had a significantly shorter head (HW) (GLM; *F* = 8.97, *P* = 0.0171; Table 3) and shorter intertegular distance (ID) (GLM; *F* = 8.00, *P* = 0.0221; Table 3) when mites were present. Again, comparisons could not be made in the DIM assay because all bee larvae died when mites were not present (Table 3).

**Table 3:** Summary of *Scaptotrigona postica* morphometric data.

Bioassay	Head width			Intertegular distance		
	CONT	TMX	DIM	CONT	TMX	DIM
Mite	332.86 ± 5.60 a	200.20 ± 0.07 b	188.00 ± 5.34	97.31 ± 2.39 b	89.55 ± 0.07 b	92.33 ± 4.58
No mite	213.83 ± 8.41 b	225.00 ± 8.00 a	NA	129.0 ± 18.44 a	145.62 ± 17.46 a	NA
df;F; > *P*	df = 1; *F* = 132.24; *P* = 0.0001	df = 1; *F* = 8.97; *P* = 0.0171		df = 1; *F* = 7.19; *P* = 0.0147	df = 1; *F* = 8.00; *P* = 0.0221	

Means followed by the same letters do not differ from each other by the t test (LSD—minimum significant difference), with Bonferroni protection (α = 0.05). *NA* No applicable.

## Discussion

Our results clearly indicate that the presence of *P*. (*N*.) *alvearii* positively increased survivorship rates when the larvae were (CONT: ca. 2.3-fold) or not exposed to pesticides (TMX: ca. 1.9-fold). The pupae and the postembrionary immatures fed on nondosed food were also favoured by the mites (ca. 1.5-fold and ca. 3.6-fold, respectively). Although the active ingredient dimethoate has both insecticide and acaricide proprieties, we observed, at least, a lower survivorship when the mites were present in all developmental phases. The reasons for these findings include the possibility of: (i) the mites playing a role in reducing the density of the fungus in the brood cells; (ii) the mites serving as a source of food for the larvae; or (iii) both possibilities occurring together.

Our findings corroborate the findings of Flechtmann and Camargo^16^ for a closely related mite species. The authors suggested that the association between *S. postica* bees and the mite *Proctotydaeus* (*Neotydeolus*) *therapeutikos* Flechtmann and Camargo was related to the removal of the fungus, which they considered to be pathogenic to the bees. They stated that nests in which *N. therapeutikos* was introduced had larval mortality reduced by almost 50%. However, a contradictory finding deserves attention: Flechtmann and Camargo’s were able to establish a fixed number of mites for each bee stage. In our study, it is unfeasible to do the same by virtue of the behaviour of the mites: *N. alverii* walk so fast among the brood cells, quickly moving cell by cell. For these reasons, likely in the present work, we are describing the behaviour of a different mite species with other kinds of roles and/or interactions. DaCosta et al.^21^ studied the mite diversity of three stingless bee species (*Melipona quadrifasciata quadrifasciata*, *Scaptotrigona bipunctata*, and *Tetragonisca fiebrigi*) in southern Brazil. The authors found that mite diversity is determined by stingless bee host species and varies according to the areas of sampling. This statement reinforces the credibility that, even for mites collected from the same bee species (*S. postica*), we found a different beneficial mite species in Flechtmann and Camargo’s study.

Currently, at least three genera of fungi proliferate in the cerumen (raw material used to build the brood combs): *Zygosaccharomyces* (required to be ingested by *Scaptotrigona* bee larvae to pupate), *Candida* (that stimulate *Zygosacchromyces* sp. development.), and *Monascus* (important ecological role)^11,12^. Our results raise the hypothesis that mites can be efficient at controlling fungal proliferation, as shown by our results with larvae exposed to TMX. This active ingredient was previously associated with remarkable changes in the development time of *Scaptotrigona depilis* immature individuals fed thiamethoxam-dosed food^22^. Additionally, other prior works reported abnormal development in honeybee larvae exposed to the same pesticide^23,24^. The progression of bee development may be affected by this neurotoxic insecticide, the fungal microbiota inside brood cells or even to control other ones, such as *Monascus*.

Under natural conditions, there is an abundant supply of fungi for both bee larvae and mites inside a brood cell. However, the fungi proliferate too fast if they are not ingested. Our hypothesis is that the bee larva become weak because of the effect posed by the pesticide (by slow feeding). Then, the fungi may proliferate at a speed that exceeds the bee larval ability to ingest it, becoming harmful for the larvae. The mites would play a cleaner role, as described by other authors^25^. This interaction would allow the larva to develop and survive instead of dying with the fungus taking over the whole cell (by suffocating and killing the larva).

This probably explains the survival rate over 33% in our treatment (TMX) with the presence of mites: as larvae are weakened by exposure to the insecticide, *Neotydeolus* ingest the fungal microbiota, helping the bees survive. In addition, the beneficial role of these mites may be observed, considering the survival rate, despite the small (ca. 8%) of larvae fed DIM-dosed food (insecticide and acaricide).

The second possibility involves mite fate soon after the larval feeding period: the mites are observed walking on and inside the brood combs, as well as within the brood cells, together with young larvae, and even on the eggs. However, as of the period preceding the total food consumption by the larvae (approximately the 4th day) up to the end of the bioassays, the mites were no longer seen or walking inside the in vitro plates, nor were they found dead (Fig. 1c). The same pattern may be observed in the natural system (Fig. 1d), in which the mites were no longer reported in older larvae (from 96 h old). This period coincides with the larval instar established by Menezes et al.^15^, in which the fungal mycelia have been eaten by stingless bee larvae.

Stingless bees and microorganisms have remarkable evolutionary relationships, as these bees rely on fermentation processes to preserve honey and store pollen^6,26–28^. De Paula et al.^14^ pointed out the three main stingless bee-microbiota associations: symbiosis, biomolecule production, and serving as food for insects. Additional studies are required to confirm our hypothesis that the N. *alvearii* mites were ingested by the larvae together with the bulk of the larval food. If this hypothesis is true, these mites would serve as a protein source or supplementary food, improving stingless bee performance, as indicated by our findings: mite presence resulting in survivorship rates of 88, 42 and 0.2% of pos-embryonic stages fed on nondosed, TMX-dosed and DIM-dosed food, respectively; without mites, the survivorship rates were, in the same sequence, 24, 27, and 0%.

Another finding that strengthens our hypothesis of the mites being ingested by the larvae raised from the development time in nondosed bees (Table 2): in the presence of mites, we observed a shorter development of the larvae but a longer development of the pupae, compared to the absence of the mites. In insects, all the food ingested by the larvae serves as a reserve of nutrients for metamorphosis, when the insect will remain without food (pupal stage). The proteins produced will serve metabolic processes in the development of the postlarval stages^29^. Pollen, being the predominant resource in the larval food of stingless bees^30^, is a protein source for them^31^. Ingested mites during the larval stage, along with the protein contained in the larval food, provide additional protein to the bees, leading them to process this additional amount. This process would influence the duration of the time for postlarval stages, explaining the longer development time in the pupae.

Vollet et al.^31^ linked the early feeding behaviour on pollen by newly emerged workers to hypopharyngeal gland development. The hypopharyngeal glands, located in the head, are involved in the production of larval food^32–34^. The size of the hypopharyngeal glands is a parameter commonly used to assess the nutritional value of food^35,36^, being larger with the increase in the amount of protein uptake^37^. As mentioned above, ingested mites provide additional protein to bees, which could result in larger hypopharyngeal glands. Measuring the size of hypopharyngeal glands was beyond the scope of our current study, but it is noteworthy that newly emerged workers fed nondosed food and associated with mites had significantly wider head capsules than those not associated with mites (a difference of ~ 119 µm wider). Workers fed TMX-dosed food and associated with mites also had a wider head capsule than those not associated with mites, but the difference was only ~ 25 µm. These findings are probably related to the neurotoxic effects of TMX, which was already proven to severely reduce bee head size^22^ and damage brain cells^38–42^.

These findings suggest that ecological and evolutionary studies should consider stingless bees, fungi and fungivorous mites together to investigate the potential role of these fungivorous mites on stingless bee health through nutrition, immune responses, and even xenobiotic metabolism. Meliponiculture (management of stingless beehives) is a promising sustainable social-economic activity in the neotropics. Weakening colonies are commonly reported by stingless beekeepers. Unlike for honeybees, knowledge about stingless bee pathogens is scarce^43–45^. A better understanding of the interactions of these organisms may optimize meliponiculture, and the manipulation of the mite in the hives can be an additional management approach to guarantee the strength of the hives.

Our work highlighted important data gaps: (i) Do brood cells with different larval stages contain different types of proliferating fungi and in different proportions? (ii) Would the mites be inside the brood cells, waiting for the proliferation of some type of fungus that could be “harmful” to the larvae before or at the same time as the *Zygosaccharomyces* proliferation, so that they can then carry out their control, thus protecting the larvae? (iii) Would the mites be important even in the control of *Zygosaccharomyces*, which could potentially proliferate at a speed that exceeds the larval ingestion capacity, killing the larvae? Moreover, these new scenarios may also encourage the search for beneficial microorganisms coexisting in other bee species, intending to mitigate the potential adverse effects from the several stressors, which may put in risk their populations. Field observations and experimental manipulations in nests of *Megalopta bees* (sweat bees), e.g., provided clear evidence of cleaning mutualisms associated with mites^25^. However, no additional investigation has been given to this issue to date. This approach may also be investigated in honeybees, since concerns on their beneficial microorganisms have been raised, requiring in-depth investigations^46^. Finally, it is essential to call attention to the worldwide practice of bee colony management, mainly for crop pollination and the potential risks of this practice. Unappropriated transport of species may facilitate the transmission of pests and diseases^47–50^. Hence, it is crucial to determine the members of communities of bee-associated organisms prevailing in different areas and the type of interaction among them so that adequate measures can be taken to prevent future problems. We expect that our findings will trigger further investigations on sustainable alternatives for healthy colonies, which may also be used to pollinate crops.

## Methods

### Provenance and husbandry of larvae

Colonies of *S. postica* originated from the experimental meliponary of the Paulista State University “Júlio de Mesquita Filho” (UNESP), which are kept in wooden hive boxes. Six nonparental colonies were used for the experiments, removing one brood comb from each. A previous standardized method for rearing stingless bee larvae was used to remove the brood combs and to obtain the larvae^17,18^.

### Provenance, husbandry and identification of mites

Mite specimens were taken from the brood combs of the native stingless bee *S. postica*, originating from the same meliponary described above. Thirty specimens were collected in 70% ethanol. The samples were collected from the same 6 nonparental colonies used for the provenance and husbandry of bee larvae (5 mites from each colony). The samples were sent to Departamento de Entomologia e Acarologia, Escola Superior de Agricultura “Luiz de Queiroz”, Universidade de São Paulo, where they were mounted in Hoyer´s medium for identification under a differential interference contrast microscope (Nikon, 80i; Nikon, Wuxi City, China).

### Behavioural observations of the mites

We used three of the 6 abovementioned brood combs to monitor mite behaviour. These combs contained bees from eggs to larvae 48 h old (younger larvae). We also collected an additional 3 brood combs from the same hives, containing larvae that were 72-h-old (older larvae). Then, we had 3 combs per group (3 replications) for carrying out the mite observations. As soon as we removed the brood combs from the hives, we placed each comb separately inside a Petri dish. In the dishes, the humidity was maintained by using wet cotton. This procedure is essential to maintain the consistency of the larval food^18^. Several humidity and temperature requirements allowed us to carry out video records no longer than 4 h. After this time, there is a quick proliferation of fungi in the brood cells. This makes further observations unfeasible.

We reported our observations through video recordings (4 h for each brood comb) and images obtained with a stereomicroscope with a coupled camera (LeicaM205 C) and LAS V4.8 software.

### In vitro bioassays

Twenty-four-hour-old larvae were transferred from their natural combs to artificial brood cells (acrylic plates) containing larval food, as described by Rosa-Fontana et al.^18^. The provenance of the larvae and the conditions of exposure were adapted from the OECD guideline for ecotoxicological bioassays on bees^51^. Six brood combs from six nonparental colonies were used. The total amount of larval food was obtained from the six colonies and homogenized in a Falcon tube before being assigned to the experimental groups. We first divided the bioassays into “no mite” (removing all the mites) and “mite” (adding *P.* (*N*.) *alvearii* mites). For the mite addition, we uncapped brood cells from brood combs containing mites; then, we added the mites by shaking pieces of brood combs onto the acrylic plates.

Immediately after the transference of larval food, larvae and mites to the acrylic plates, we checked all the plates in a stereomicroscope with a coupled camera (LeicaM205 C) and LAS V4.8 software. The mites are easily visible in these conditions, and then we assured the “no mite” and “mite” bioassays. Each bioassay was subdivided into experimental groups: larvae fed (i) thiamethoxam-dosed food (TMX), (ii) dimethoate-dosed food (DIM), and (iii) control (CONT). The control group (negative control) consisted of larvae fed pure food, with no pesticide addition. From each of the 6 colonies, 60 larvae were sampled, and 20 of them were randomly assigned to the different experimental groups. Each experimental group contained 3 replicates (each replicate was represented by 1 acrylic plate containing 20 larvae). Then, each experimental group consisted of 60 larvae (180 larvae/bioassay).

### Preparation of larval food with pesticides

The active ingredients (a. i.) (Pestanal, analytic standard, Sigma-Aldrich, Burlington, MA, USA) thiamethoxam (TMX) and dimethoate (DIM) were offered to the larvae through the food. Doses were based on the field recommendations, considering bee attractive crops, registered in the “Ministry of Agriculture, Livestock and Supply” (AGROFIT). To estimate the amount of residue in nectar and pollen, the recommended dose for the field was plotted in the Bee Rex table proposed by the United States Environmental Protection Agency (USEPA). We diluted a stock solution of 1000 ng a.i./µL of food at 1:1 (10 mg of TMX or DIM: 10 mL of larval food) to reach the experimental concentrations of 0.00157 ng a.i./µL (TMX) and 0.007 ng ia/µL (DIM). These concentrations represent field realistic doses (FDR): FDR/1000 (TMX) and FDR/100 (DIM). The active ingredient was diluted directly into larval food to reach an initial concentration of 1000 ng a.i./µL, according to an established protocol (Dorigo et al.^17^). As the amount of food required in each brood cell was 25 µL, we multiplied each value by 25 to obtain the concentration in ng a. i./larva: 0.03925 (TMX) and 0.175 (DIM). Stingless bees massively feed their larvae; that is, they deposit the total amount of food at once in the breeding cells, which will be completely consumed by the larvae^17,18^. This massive system enables us to determine the total concentration ingested by bees.

### Survivorship, development, and morphometric measurements of *S. postica* larvae assessments

The in vitro plates were monitored daily for mortality and developmental time. Dead individuals were removed from the experiments. The intertegular distance and head width were used as morphometric parameters for checking possible variations among the bioassays with and without mites. Therefore, from each experimental group (as described in “in vitro bioassays”), 30 bees were used for the morphometric assessments. The measurements were made by using images obtained with a stereomicroscope with a coupled camera (Leica M205 C) and LAS V4.8 software using the measurement module of the software itself.

### Statistical analysis

We used a factorial structure to analyse the interaction involving treatments (insecticides and control) versus mite effect (absence and presence of the mites) in the following variables: survivorship, duration time of development, head width and intertegular distance. Therefore, we compared the mite effect in relation to its absence within each treatment. We used a generalized linear model with quasibinomial distribution to compare the survival rate, and the model chosen here to fit the survival data was carefully selected based on their goodness-of-fit using residual plots and half-normal plots^52^. The variables duration of development, head width and intertegular distance were analysed with pairwise Bonferroni’s t-test (protected least significant difference (LSD)) comparisons between group levels (α = 0.05). Multiple comparisons of survival curves and pairwise comparisons between group levels with corrections for multiple testing were performed with the packages survminer^53^ and survival^54^ in R software.

## Supplementary Information

Supplementary Video 1.

Supplementary Information 1.

## Data Availability

All data generated or analysed during this study are available from the corresponding author on reasonable request.
