# Supplementary material for: Fungivorous mites enhance the survivorship and development of stingless bees even when exposed to pesticides
Source: Sci Rep. 2022 Dec 5;12:20948. doi: 10.1038/s41598-022-25482-x (PMC9722777; doi:10.1038/s41598-022-25482-x)
Supplement: Supplementary file 2 — Supplementary Information 1. [file 41598_2022_25482_MOESM2_ESM.docx]

**Supplementary Video 1. Brood comb of *Scaptotrigona postica*.** The brood comb shows the coexistence between the bee larvae and the *Proctotydaeu*s (*Neotydeolus*) *alverii* mites. The small white points moving quickly are the mites: they move within and among the brood cells.
